# Supplementary material for: Association between Gingival Phenotype and Periodontal Disease Severity—A Comparative Longitudinal Study among Patients Undergoing Fixed Orthodontic Therapy and Invisalign Treatment
Source: Healthcare (Basel). 2024 Mar 14;12(6):656. doi: 10.3390/healthcare12060656 (PMC10970162; doi:10.3390/healthcare12060656)
Supplement: Supplementary file 1 [file healthcare-12-00656-s001.zip › healthcare-2900624-supplementary.pdf]

Table S1: Comparison of clinical parameters between three groups (thick phenotype)

Thick phenotype		N	Mean	Std. Deviation	Std. Error	95% Confidence Interval for Mean		p-value
						Lower Bound	Upper Bound	
**Transgingival probing (mm)**	Invisalign	15	1.6467	.27220	.07028	1.4959	1.7974	0.001
	FOT	15	1.7067	.27115	.07001	1.5565	1.8568	
	Control	15	1.2394	.28963	.07478	1.0790	1.3998	
	Total	45	1.5309	.34311	.05115	1.4278	1.6340	
**KTW**	Invisalign	15	5.2067	.23135	.05973	5.0785	5.3348	0.039
	FOT	15	6.0925	1.51929	.39228	5.2511	6.9339	
	Control	15	4.9072	1.58132	.40830	4.0315	5.7830	
	Total	45	5.4021	1.34392	.20034	4.9984	5.8059	
**FMPS (baseline)**	Invisalign	15	18.0413	7.96330	2.05612	13.6314	22.4513	0.038
	FOT	15	14.4483	8.95547	2.31229	9.4889	19.4076	
	Control	15	10.9243	4.11042	1.06131	8.6480	13.2006	
	Total	45	14.4713	7.72694	1.15186	12.1499	16.7927	
**FMPS (6 months)**	Invisalign	15	42.9120	19.28847	4.98026	32.2304	53.5936	0.001
	FOT	15	26.7971	14.16178	3.65656	18.9546	34.6397	
	Control	15	9.9197	2.11269	.54549	8.7497	11.0896	
	Total	45	26.5429	19.21415	2.86428	20.7704	32.3155	
**Gingival index (baseline)**	Invisalign	15	.4287	.13094	.03381	.3562	.5012	0.348
	FOT	15	.5436	.34680	.08954	.3515	.7356	
	Control	15	.5775	.33974	.08772	.3894	.7656	
	Total	45	.5166	.29086	.04336	.4292	.6040	
**Gingival index (6 months)**	Invisalign	15	1.2622	.65211	.16837	.9011	1.6233	0.01
	FOT	15	1.6477	.77722	.20068	1.2173	2.0781	
	Control	15	.8962	.45692	.11798	.6432	1.1492	
	Total	45	1.2687	.70016	.10437	1.0583	1.4791	
**FMBS (baseline)**	Invisalign	15	7.7684	4.45205	1.14951	5.3029	10.2338	0.313
	FOT	15	6.8299	3.18426	.82217	5.0665	8.5932	
	Control	15	9.0140	3.91180	1.01002	6.8478	11.1803	
	Total	45	7.8708	3.90131	.58157	6.6987	9.0428	
**FMBS (6 months)**	Invisalign	15	16.8714	5.66551	1.46283	13.7339	20.0088	0.016
	FOT	15	19.4878	6.02037	1.55445	16.1538	22.8218	
	Control	15	13.6740	3.89990	1.00695	11.5143	15.8337	
	Total	45	16.6777	5.68905	.84807	14.9685	18.3869	
**PD (baseline)**	Invisalign	15	1.9940	.11070	.02858	1.9327	2.0553	0.001
	FOT	15	1.2880	.26444	.06828	1.1416	1.4344	
	Control	15	1.1869	.18063	.04664	1.0869	1.2869	
	Total	45	1.4896	.41031	.06117	1.3664	1.6129	
**PD (6 months)**	Invisalign	15	3.0067	.29391	.07589	2.8439	3.1694	0.001
	FOT	15	2.3134	.33464	.08640	2.1281	2.4988	
	Control	15	1.3555	.24806	.06405	1.2181	1.4929	
	Total	45	2.2252	.74256	.11069	2.0021	2.4483	
**CAL (baseline)**	Invisalign	15	.9931	.55129	.14234	.6878	1.2984	0.481
	FOT	15	1.0237	.46403	.11981	.7667	1.2806	
	Control	15	.8404	.25093	.06479	.7015	.9794	
	Total	45	.9524	.43797	.06529	.8208	1.0840	

**CAL (6 months)**	Invisalign	15	1.0720	.44685	.11538	.8245	1.3194	0.182
	FOT	15	1.0038	.33751	.08714	.8169	1.1907	
	Control	15	.8061	.41251	.10651	.5777	1.0346	
	Total	45	.9606	.40856	.06090	.8379	1.0834	
**GR (baseline)**	Invisalign	15	.8520	.29459	.07606	.6888	1.0151	0.001
	FOT	15	1.5365	.83441	.21544	1.0744	1.9986	
	Control	15	.7089	.37948	.09798	.4988	.9191	
	Total	45	1.0325	.65450	.09757	.8358	1.2291	
**GR (6 months)**	Invisalign	15	1.2249	.34950	.09024	1.0313	1.4184	0.001
	FOT	15	2.0338	.85118	.21977	1.5624	2.5052	
	Control	15	.8906	.37881	.09781	.6808	1.1004	
	Total	45	1.3831	.74204	.11062	1.1601	1.6060	

FMPS: full mouth plaque score; FMBS: full mouth bleeding score; GI: gingival index; PPD: probing pocket depth; CAL: clinical attachment loss; GR: gingival recession; KTW: keratinized tissue width; FOT: fixed orthodontic treatment; one-way ANOVA p<0.05: significant

Table S2: Comparison of clinical parameters between three groups (thin phenotype)

Thin phenotype		N	Mean	Std. Deviation	Std. Error	95% Confidence Interval for Mean		p-value
						Lower Bound	Upper Bound	
**Transgingival probing (mm)**	Invisalign	15	.8480	.17215	.04445	.7526	.9433	0.001
	FOT	15	.5533	.21668	.05595	.4333	.6733	
	Control	15	.8900	.02661	.00687	.8752	.9047	
	Total	45	.7638	.21803	.03250	.6983	.8293	
**KTW**	Invisalign	15	4.2370	1.18290	.30542	3.5819	4.8921	0.017
	FOT	15	5.3776	1.68732	.43566	4.4432	6.3120	
	Control	15	4.0407	.97128	.25078	3.5028	4.5785	
	Total	45	4.5517	1.41654	.21117	4.1262	4.9773	
**FMPS (baseline)**	Invisalign	15	11.2040	3.14563	.81220	9.4621	12.9460	0.001
	FOT	15	6.1800	.41420	.10695	5.9506	6.4094	
	Control	15	8.2961	.33086	.08543	8.1129	8.4794	
	Total	45	8.5601	2.75243	.41031	7.7331	9.3870	
**FMPS (6 months)**	Invisalign	15	25.0478	4.43740	1.14573	22.5905	27.5052	0.001
	FOT	15	18.1469	5.83999	1.50788	14.9128	21.3810	
	Control	15	11.8213	4.01407	1.03643	9.5984	14.0442	
	Total	45	18.3387	7.21679	1.07582	16.1705	20.5068	
**Gingival index (baseline)**	Invisalign	15	.8675	.21548	.05564	.7481	.9868	0.001
	FOT	15	.6543	.14382	.03713	.5746	.7339	
	Control	15	1.2135	.55753	.14395	.9048	1.5223	
	Total	45	.9118	.41782	.06228	.7862	1.0373	
**Gingival index (6 months)**	Invisalign	15	1.7471	.33153	.08560	1.5635	1.9307	0.001
	FOT	15	1.4271	.25383	.06554	1.2865	1.5677	
	Control	15	2.4276	.39614	.10228	2.2082	2.6469	
	Total	45	1.8673	.53235	.07936	1.7073	2.0272	
**FMBS (baseline)**	Invisalign	15	16.5894	5.55201	1.43352	13.5148	19.6640	
	FOT	15	9.8297	4.29475	1.10890	7.4513	12.2080	
	Control	15	16.6221	4.36326	1.12659	14.2058	19.0384	
	Total	45	14.3470	5.67183	.84551	12.6430	16.0510	

**FMBS (6 months)**	Invisalign	15	35.4750	11.21124	2.89473	29.2664	41.6836	0.005
	FOT	15	25.3229	5.56867	1.43782	22.2391	28.4067	
	Control	15	28.5149	6.69511	1.72867	24.8073	32.2225	
	Total	45	29.7709	9.08277	1.35398	27.0422	32.4997	
**PD (baseline)**	Invisalign	15	2.0428	.56153	.14499	1.7319	2.3538	0.048
	FOT	15	1.9266	.50891	.13140	1.6448	2.2084	
	Control	15	1.5441	.60117	.15522	1.2112	1.8770	
	Total	45	1.8378	.58665	.08745	1.6616	2.0141	
**PD (6 months)**	Invisalign	15	1.7745	.70680	.18250	1.3831	2.1659	0.001
	FOT	15	2.7614	.33938	.08763	2.5735	2.9494	
	Control	15	1.9777	.52272	.13497	1.6882	2.2672	
	Total	45	2.1712	.68390	.10195	1.9657	2.3767	
**CAL (baseline)**	Invisalign	15	2.0428	.56153	.14499	1.7319	2.3538	0.67
	FOT	15	1.9952	1.16289	.30026	1.3512	2.6392	
	Control	15	1.7910	.56892	.14689	1.4760	2.1061	
	Total	45	1.9430	.80361	.11980	1.7016	2.1844	
**CAL (6 months)**	Invisalign	15	1.7745	.70680	.18250	1.3831	2.1659	0.001
	FOT	15	3.3116	1.14359	.29527	2.6783	3.9449	
	Control	15	1.6761	.56800	.14666	1.3615	1.9906	
	Total	45	2.2540	1.11860	.16675	1.9180	2.5901	
**GR (baseline)**	Invisalign	15	.7708	.25990	.06711	.6268	.9147	0.169
	FOT	15	.7936	.43187	.11151	.5545	1.0328	
	Control	15	1.1260	.83955	.21677	.6611	1.5909	
	Total	45	.8968	.57625	.08590	.7237	1.0699	
**GR (6 months)**	Invisalign	15	1.3976	.72317	.18672	.9971	1.7980	0.158
	FOT	15	1.8337	.55722	.14387	1.5251	2.1423	
	Control	15	1.4861	.63720	.16452	1.1332	1.8389	
	Total	45	1.5724	.65622	.09782	1.3753	1.7696	

FMPS: full mouth plaque score; FMBS: full mouth bleeding score; GI: gingival index; PPD: probing pocket depth; CAL: clinical attachment loss; GR: gingival recession; KTW: keratinized tissue width; FOT: fixed orthodontic treatment; one-way ANOVA $p<0.05$: significant
